# Per-Span Microwave-Frequency Fiber Interferometry for Amplified Transmission Links Employing High-Loss Loopbacks

**DOI:** 10.3390/s26082551

**Published:** 2026-04-21

**Authors:** Georgios Aias Karydis, Menelaos Skontranis, Christos Simos, Iraklis Simos, Thomas Nikas, Charis Mesaritakis, Adonis Bogris

**Affiliations:** 1 Department of Informatics and Computer Engineering, University of West Attica, Aghiou Spiridonos, Egaleo, 12243 Athens, Greece; gakaryidis@uniwa.gr; 2 Department of Biomedical Engineering, University of West Attica, Aghiou Spiridonos, Egaleo, 12243 Athens, Greece; mskontranis@uniwa.gr (M.S.); cmesar@uniwa.gr (C.M.); 3 Electronics & Photonics Laboratory, Department of Physics, University of Thessaly, 35100 Lamia, Greece; christos.simos@uth.gr; 4 Department of Electrical and Electronics Engineering, University of West Attica, Aghiou Spiridonos, Egaleo, 12243 Athens, Greece; simos@uniwa.gr; 5 Department of Informatics and Telecommunications, National and Kapodistrian University of Athens, 15784 Athens, Greece; tnikas@di.uoa.gr

**Keywords:** fiber-optic sensing, optical interferometry, submarine cables

## Abstract

**Highlights:**

**What are the main findings?**

- We propose and experimentally demonstrate a variant of a microwave-frequency fiber interferometer than can support multi-span–multi-loop interrogation in a two-loop proof-of-principle experiment.
- We theoretically estimate the efficacy of the technique in comparison to optical interferometric schemes based on state-of-the-art fiber lasers and reveal that per-span interferometry based on microwave frequencies can provide better detectivity at low frequencies (<1 Hz), where most of the critical geophysical events manifest.

**What are the implications of the main findings?**

- The analysis shows that a properly engineered microwave-frequency fiber interferometer is worth considering when investigating ultra-long fiber links using high-loss loopbacks.
- The analysis shows that the specific system has a clear advantage in detecting low-frequency events, achieving a better signal-to-noise ratio, when compared to optical interferometric systems based on state-of-the-art fiber lasers.

**Abstract:**

The use of long-distance transoceanic cables equipped with high-loss loopbacks enables distributed sensing with a resolution determined by amplifier spacing, typically in the order of 50–100 km. Microwave-frequency fiber interferometry is a promising trans-mission technique for investigating long links supported by periodic optical amplification. In this paper, we propose a variant of this technique that ensures compatibility with links containing high-loss loopbacks, thereby transforming the integrated sensing approach into a distributed one. We highlight the critical modifications required to overcome challenges associated with the detection of multiple return signals, and we conduct a proof-of-principle experiment using a two-loop configuration. We demonstrate the concept by detecting and localizing low-frequency (<10 Hz) events—whether human-generated or induced by fiber stretchers—with span-level resolution. This validates the potential of the modified microwave-frequency interferometry approach for transoceanic cable monitoring in scenarios where high-loss loopbacks are present. We also present a theoretical analysis that evaluates the limits of the technique across different frequency ranges, in comparison with optical interferometry methods based on high-spectral-purity fiber lasers. The analysis shows that for long amplifier spacings (~100 km), micro-wave-frequency fiber interferometry exhibits a signal-to-noise ratio advantage at sub-Hz frequencies (<0.1 Hz) compared to state-of-the-art optical interferometers.

## 1. Introduction

Fiber-optic sensing techniques integrated into already-installed cable networks have gained significant traction over the past decade due to their wide range of potential applications, spanning optical network surveillance, smart city systems, and geophysical and environmental monitoring [1,2,3]. In particular, geophysical monitoring via fiber-optic sensing offers a powerful new opportunity, enabled by the global network of submarine cables, which provide a sensing platform in remote areas where traditional instruments are difficult to deploy. Distributed acoustic sensing (DAS) in the form of coherent or phase optical time-domain reflectometry is a mature, high-sensitivity (picostrain level) and high-resolution (sub-meter) fiber interrogation technique [4]. The reach of state-of-the-art commercially available DAS systems support is limited to 150 km at most in commercially available variants [5]. Nevertheless, its use in long-haul submarine cables carrying network traffic is questionable because DAS is less compatible with periodic amplification and it emits a high amount of power per pulse (>100 mW), which can induce nonlinearities. Despite recent advancements based on coding and bidirectional amplification [6,7], DAS variants are still immature for deployment in long-haul amplified links.

The interrogation of long-haul links has instead been successfully demonstrated using interferometric techniques [8] and polarization-state measurements [9]. Early demon-strations treated multi-thousand-kilometer links as single sensors, offering limited localization capabilities. However, a more recent approach [10] introduced a different perspective by leveraging existing high-loss loopbacks (HLLBs) to transform a single long link into an array of sensors, with spatial resolution determined by the periodic placement of optical amplifiers. In this configuration, the link enables localization with a resolution of 50–100 km, which is sufficient for detecting and locating geophysical events such as earthquakes, ocean swells, and tsunamis [10].

Such geophysical phenomena are characterized by long wavelengths (up to hundreds of kilometers); therefore, the meter-scale spatial resolution offered by DAS is generally unnecessary, particularly for early-warning applications [11]. Since then, several additional demonstrations have employed optical interferometry, coherent optical frequency-domain reflectometry, and polarization-based techniques that exploit high-loss loopbacks in installed transoceanic cables, aiming to transform these infrastructures into global seismic monitoring networks [12,13,14].

A technique compatible with long-haul cable interrogation is the so-called micro-wave-frequency fiber interferometry (MFFI) [15]. Its theoretical foundations and effectiveness in earthquake detection have been demonstrated in both terrestrial and sub-marine installations [15,16]. This technique offers distinct advantages in terms of simplicity and cost, as it replaces high-quality, narrow-linewidth lasers with off-the-shelf microwave oscillators that exhibit superior spectral purity in absolute terms (sub-Hz linewidth), enabling sub-millihertz response—an essential requirement for geophysical monitoring [15]. Recent experiments in Cephalonia, Greece, have shown that MFFI can detect undersea microearthquakes (magnitude ~1.5), as well as low-frequency phenomena such as ocean waves [16], demonstrating sufficient sensitivity, particularly at frequencies below 0.1 Hz. This capability, combined with its low-cost implementation, makes MFFI a strong can-didate for large-scale deployment in early-warning systems for natural hazards in the deep ocean. More recently, a digital variant of MFFI was presented in [17], enabling distributed measurements over a transatlantic cable connecting Portugal and Brazil. In this approach, phase measurements are performed in the digital domain using a 90 MHz microwave modulation. The scheme can provide low-frequency seafloor pressure and temperature monitoring. The authors also state that the signal-to-noise ratio can be improved by using a higher frequency carrier (10 GHz and above). At high RF frequencies, the digital acquisition of phase changes will be more challenging in terms of hardware and data pre-processing (>20 Gsa/s sampling rate for 10 GHz carriers will be needed). MFFI implementations of [15,16] use the same low-noise PLL-based oscillator (−90 dBc/Hz @ 100 Hz offset frequency) at both the transmitter and the receiver. Phase discrimination is carried out in the analog domain, generating baseband phase signals that can be digitized using low-bandwidth analog-to-digital converters (sub-MSa/s), independent of the RF carrier frequency.

In the present work, we present a variant of MFFI that is compatible with the interrogation of long-haul links assisted by periodic loopbacks. We set up a proof-of-principle experiment in a 70 km link consisting of two spans. One of the spans was a real-life 30 km long loop connecting our lab with the municipality of Piraeus. The second was a 40 km long fiber spool residing in the lab. By applying mechanical vibrations induced by either a commercial fiber stretcher or by human activity, we verified that the modified detection scheme could distinguish events on a per-span basis with high stability and a high signal-to-noise ratio. We also conducted a theoretical analysis that clearly shows how the specific interferometer compares to its optical counterpart, taking into account the phase noise properties of state-of-the-art microwave and fiber-laser sources, along with the accumulated amplified spontaneous emission noise introduced by inline amplifiers. The analysis clearly shows that the microwave version of fiber interferometry exhibits a higher signal-to-noise ratio at the low-frequency regime (<0.1 Hz).

## 2. Materials and Methods

### 2.1. Basic Principle of Per-Span MFFI

**Figure 1:**
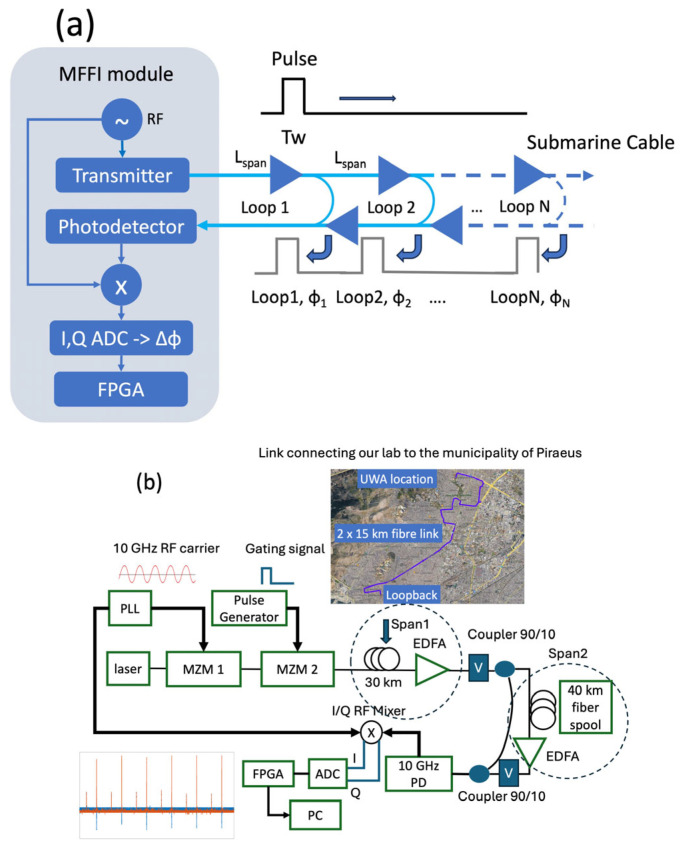
(**a**) Conceptual scheme of MFFI in pulsed mode for interrogating submarine cables employing high-loss loopbacks. (**b**) Experimental setup of the MFFI sensing system operating with periodic loopbacks. PLL: phase-locked loop, MZM: Mach–Zehnder modulator, PD: photodiode, EDFA: erbium-doped fiber amplifier, ADC: analog-to-digital converter, FPGA: field-programmable gate array, PC: personal computer.

The basic idea of per-span interferometry using MFFI is depicted in Figure 1a. The phase changes detected by MFFI are expressed as $\varphi =\frac{2\pi f_{RF}n_{g}L}{c}$, where *f_rf_* is the radio frequency, *n_g_* is the refractive index and *L* is the round-trip time of the interferometer [15]. When a multi-loop system is used, the phase detected by the *i*-th return signal is connected to that of the (*i*−1)th span through the following equation: $\varphi_{i}=\varphi_{i-1}+\frac{2\pi f_{RF}n_{g}2L_{span}}{c}$. Hence, the induced phase changes over the *i*-th span can be calculated as ${\Delta \varphi }_{i}=\varphi_{i}-\varphi_{i-1}$. In this way, one can easily identify the strain or temperature variations affecting each span. In order to interrogate each span without experiencing any interference of crosstalk from other spans, the MFFI must be operated in pulsed mode, as depicted in Figure 1a. A pulse with a width *T_w_* that is less than the round trip per span ($T_{w}<\frac{n_{g}2L_{span}}{c}$) must be sent with a repetition period *T_rep_* of the time required to interrogate the total number of spans, specifically $T_{rep}>\frac{n_{g}2NL_{span}}{c}$ [14,17]. The duty cycle of the pulse must be less than 1/*N* where *N* is the targeted number of spans to be interrogated. The maximum sampling frequency per span is limited by *T_rep_* (*f_s_* < 1/*T_rep_*). The next paragraph describes the experimental setup.

### 2.2. Experimental Setup and Data Acquisition

The modified MFFI configuration is depicted in Figure 1b. Its main differences compared to the scheme presented in [13] are the use of pulsed operation and the in-corporation of an I/Q RF mixer instead of a conventional RF mixer. In fiber-optic sensing applications, an acousto-optic modulator is typically used to provide strong gating. In our implementation, optical gating is achieved using a second-intensity Mach–Zehnder modulator. The inclusion of an I/Q RF mixer is another key enhancement, enabling the simultaneous acquisition of both quadratures. This ensures maximum sensitivity across all loopbacks concurrently. In previous implementations [15,16], only the in-phase (I) component was detected, and a microwave phase shifter was used to actively control the relative phase between the signal and the local oscillator, maintaining operation at the quadrature point for optimal sensitivity. While this approach is effective when interrogating the entire link as a single sensor, it is not compatible with the parallel interrogation of multiple loopbacks in the per-span interferometry framework. This limitation arises because the required phase offset for operation at the quadrature point differs from span to span.

The full experimental setup comprises a tunable laser, operating at 1550 nm and serving as the light source for the optical path in our experimental setup. The laser output is directed into two cascaded-intensity Mach–Zehnder modulators (MZMs). The first modulator is directly driven by a phase-locked loop (PLL) to modulate the optical intensity at 10 GHz. The second modulator, controlled by a pulse generator, plays the role of the gating module producing light pulses. The light pulses are then fed into an optical link comprising two spans. The first span consists of a 30 km segment of an installed cable connecting the municipality of Piraeus to our lab. This cable is deployed in a densely populated area of Athens and records significant levels of anthropogenic noise, including car traffic, construction activity, and pedestrian movement, thereby enhancing the reliability of our findings. The second span consists of a 40 km fiber spool. The pulse duration must be less than the propagation delay induced by the second loop, which is approximately 0.2 ms. Here, we use a pulse duration from 40 μs to 100 μs and a repetition period of 500 μs. To amplify the optical pulses, two erbium-doped fiber amplifiers (EDFAs) are used—one per span. The first coupler sends 90% of power to the next loop, and 10% of the power is looped back to the receiver. The second coupler directs 90% of the signal from loop 1 and 10% from loop 2 to the receiver. The losses incurred per loop due to the couplers are approximately 10.45 dB. A typical high-loss loopback (HLLB) system introduces at least 30 dB of loss at the reflected wavelength [10]. In our setup, the total loss is further increased by omitting an additional erbium-doped fiber amplifier (EDFA) prior to photodetection, which is commonly included in experiments employing HLLBs for fiber-sensing applications [10,12]. The propagation and splicing losses of the two links shown in Figure 1 are 9 dB and 17 dB, respectively. The gains of EDFA1 and EDFA2 were carefully adjusted, and the optical powers of the two loops were measured to be nearly equal after being combined in the second coupler (approximately −15 dBm each). The combined optical signal is then detected by a 10 GHz photodiode (PD), which converts it into an RF signal that serves as one input to the I/Q RF mixer. The second input is a locally generated 10 GHz signal, which acts as the local oscillator (LO). 

The two intermediate-frequency (IF) outputs (I and Q) of the mixer are driven to a 16-bit ADC, operating in a sampling rate of 1 MSa/s. Samples are stored to a PC in real time through a field programmable gate array (FPGA), which is programmed to average the signals and perform downsampling to 64 ksa/s. The phase noise psd of the original MFFI has been reported in [15], and was found to be below −60 dBc/Hz at 0.1 Hz, which clearly shows the potential of the technique for low-noise measurements at low frequencies, even reaching the sub-mHz range, which is an important frequency window for geophysical monitoring. We measured the phase noise in the new setup using a fiber interferometer with 1 km delay and found that it exhibits even better noise performance (−80 dBc/Hz at 0.1 Hz, −90 dBc/Hz at 1 Hz) as a result of I/Q detection, which offers a higher signal-to-noise ratio and a better ADC device at the reception (16 bit, 1 Msa/s) compared to [15]. These results will be better highlighted in Section 3. The next paragraph provides the basic elements of our theoretical approach.

### 2.3. Theoretical Approach for Phase Noise Estimation

Apart from a proof-of-concept experiment, we theoretically examine the efficiency of such a scheme in a submarine long-haul transmission system in terms of the phase noise power spectral density (PSD), which is a valid metric used to identify the capabilities of the sensing technique in detecting events in diverse frequency windows. Our theoretical analysis takes into account two main noise sources, namely the noise of the oscillator and the amplified spontaneous emission noise that is accumulated along the link. Per-span interferometry has been presented along with the use of very stable laser sources [10,12]. The alternative is to use a microwave oscillator as proposed in this work. The phase noise of the source is usually known a priori and provided by the manufacturer. In the case of MFFI, the microwave source (PLL) is characterized by *S_φ,PLL_*(*f*) noise PSD. In the case of optical interferometry, a fiber laser is usually employed and characterized by *S_φ,FL_*(*f*). The interferometer provides a high correlation at low frequencies; therefore, the overall noise PSD resulting from the interplay between source phase noise and interferometer is given by the following expression:

(1) $$S_{\varphi s,int}(f)=S_{s\varphi }\left(f\right){\left|1-\exp\left(-i2\pi f{Τ}_{d}\right)\right|}^{2}$$

where *S_sφ_* is the phase noise PSD of the high-spectral-purity source (microwave or laser-based oscillator), and *T_d_* is the delay induced by the long arm of the interferometer. This is related to the transmission distance *T_d_* = *n_g_L*/*c*, where c is the speed of light and *n_g_* is the refractive index of the optical fiber, which, for this analysis, can be considered to be equal to *n_g_* = 1.5.

The noise related to the optical signal-to-noise ratio of the received signal provides a spectrally uniform phase noise PSD which can be expressed as $\frac{1}{ESNR*{2B}_{el}}$, where *ESNR* is the electrical signal-to-noise ratio (SNR) after the photodetection, and 2*B_el_* is the two-sided electrical bandwidth considered for ESNR measurement, assuming that the main noise term at the photodetection for such a long-haul link is the signal-ASE beating. If *N_amp_* amplifiers are used in a long-haul link, then the ASE PSD is provided by the well-known expression $S_{ASE}=2N_{amp}n_{sp}hf_{opt}\left(G-1\right)$, considering that the gain *G* of each amplifier perfectly compensates for the span losses, with *h* being Planck’s constant, *f_opt_* the optical frequency (around 190 THz), and *n_sp_* the spontaneous emission factor. The expression that describes the signal-ASE beating dominated ESNR is:

(2) $$ESNR=\frac{P_{s}}{2S_{ASE}B_{el}}$$

In (2), *P_s_* is the optical power that reaches the photodetector. Based on (2), one can easily identify the corresponding phase noise PSD term as follows:

(3) $$S_{\varphi ,ASE,OFI}(f)=\frac{S_{ASE}}{P_{s}}$$

where *S_ASE_* is the accumulated PSD of the noise and *P_s_* is the power of the signal of interest. In the case of MFFI, the optical carrier is modulated in intensity and expressed as $P\left(t\right)=P_{s}\left[1+mcos\left(2\pi f_{RF}t\right)\right]$, where *m* is the modulation depth and *f_RF_* is the microwave frequency, which in this work is considered to be 10 GHz. Hence, the electrical power of the 10 GHz signal after photodetection is scaled by a factor of *P_s_^2^m*^2^/4 (single-sideband power), while signal-ASE beating is dominated by the average power of the optical signal, which is *P_s_* as before. Therefore, the phase noise PSD for MFFI can be expressed as follows:

(4) $$S_{\varphi ,ASE,MFFI}(f)=\frac{{4S}_{ASE}}{P_{s}m^{2}}$$

Using the aforementioned formulas, one can extract useful information about the performance of microwave-frequency fiber interferometry and optical interferometry in long-haul transmission schemes employing high-loss loopbacks. More details on the theoretical model are presented in Section 3.

## 3. Results

### 3.1. Experimental Results

**Figure 2:**
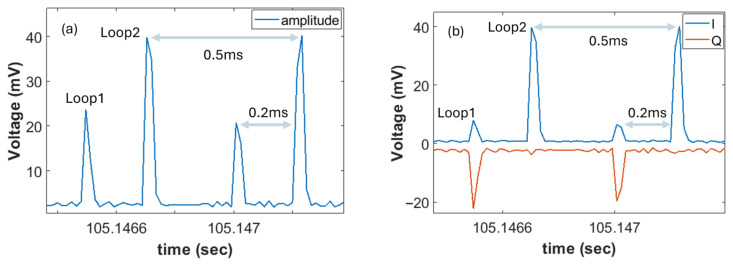
Snapshot from the timetraces of the amplitude (**a**) and I andQ components (**b**). The blue line corresponds to the I component, whilst the red one corresponds its Q counterpart. The delay of 0.2 ms corresponds to the propagation delay induced by the 40 km spool (2nd span). The periodicity of 0.5 ms corresponds to the 2 kHz of the gating repetition rate.

In order to evaluate the detection and localization capabilities of the system, we apply controlled vibrations either at span 1 or span 2. These vibrations were created by manually and loosely shaking or bending specific patch cords belonging to the two links and with the use of a fiber stretcher. In principle, if one applies a vibration to the first link, then this vibration will affect the returned pulses of both spans (*φ*_1_ and *φ*_2_). On the contrary, a vibration applied to the second span will only appear to the second loop pulses (*φ*_2_). Before showing the vibrations in each case, the time trace of the amplitude and I/Q components for both loops are depicted in Figure 2a and Figure 2b, respectively. The time difference of approximately 0.2 ms is the propagation delay induced by span 2 (40 km). The 0.5 ms periodicity corresponds to the 2 kHz repetition rate of the gating. The sign and magnitude of I/Q components may change over time as a result of the phase experienced by the 10 GHz signal after the mixing process. Each loop will experience its own integrated phase due to propagation (*φ_i_*). The recovery of both I and Q components provide the possibility of extracting the phase signal per loop at the receiver. The lower amplitude observed in loop 1 is attributed to chromatic dispersion induced power fading. This can be easily verified by calculating the dependence of power fading as a function of distance for the 10 GHz tone propagating in typical single-mode fibers with a dispersion of 16–17 ps/nm/km in the C-band. Based on the well-known expression of power fading which shows that the detected RF power is proportional to $\cos^{2}\left({2\pi^{2}\beta }_{2}f_{RF}^{2}L\right)$, we can easily identify that the loop 1 RF component (~30 km) could be 7.5–10 dB less powerful than the loop 2 component (~70 km) in the RF domain. In the voltage level, this translates to 3.5 to 5 dB, which seems to be verified in Figure 2a, with a 1 dB declination attributed to a small power imbalance between the two loops.

**Figure 3:**
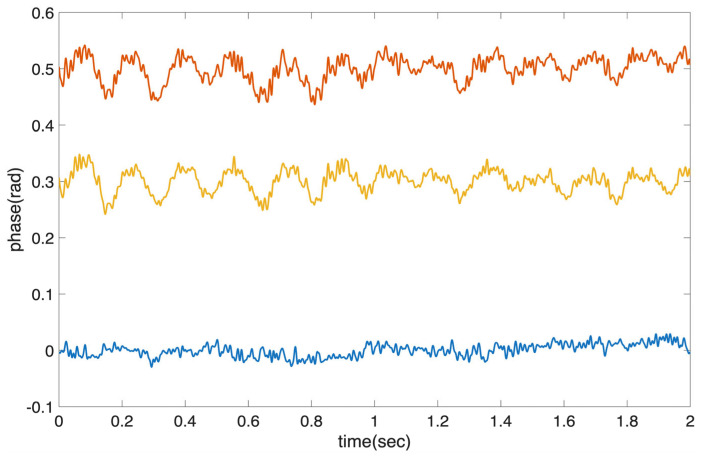
Loop 2 and loop 1 phase signals and their differences, which correspond to vibrations induced in the 40 km spool through loose tensions of one patchcord.

**Figure 4:**
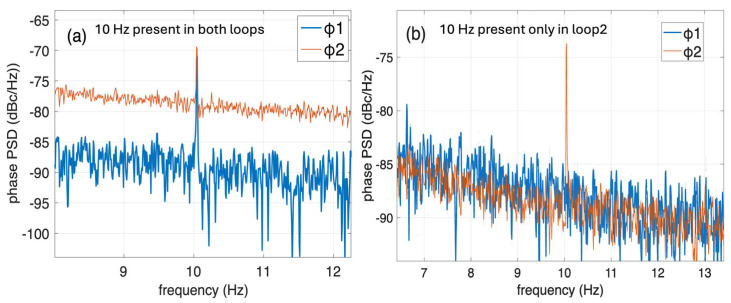
Power spectra density of loop 1 and loop 2 phase signals when (**a**) loop 1 or (**b**) loop 2 is under a fiber stretcher vibration.

First, we apply a hand-made loose tension on a patch-cord belonging to the second span (40 km spool). Then, we record the data and separate the signals of loop 1 and loop 2. We retrieve the phase per loop based on the I/Q components depicted in Figure 2. A time trace of the phase over time shown in Figure 3 clearly shows that the aforementioned vibration is evident only in the loop 2 signal, which integrates the vibrations of both spans. The difference in *φ*_2_ − *φ*_1_, shifted on purpose to 0.3 rad in order to be easily distinguished from the loop 2 signal, clearly shows that the vibration has solely affected the second span, as expected. We also tested a controllable vibration using the fiber stretcher and applied a sinusoidal signal of 10 Hz at a voltage of 2 V, corresponding to maximum elongation of 5 μm. We select 10 Hz, which is the minimum frequency supported by our stretcher, rather than a higher-frequency stimulus in the kHz range, because geophysical effects such as earthquakes, ocean swells, and tsunamis exhibit significant frequencies between 0.01 Hz and 10 Hz [10,11,12,13,14,15,16]. Tsunamis reside at even lower frequencies (mHz) [7,11]. Figure 4 shows the power spectral density of the phase signals of loop 1 and loop 2 in dBc/Hz (single sideband). It is evident that when the vibration is located in span 1, it can be seen in both loop 1 and loop 2 spectra. On the contrary, when the vibration element is within span 2, the 10 Hz signal becomes visible only in the loop 2 spectrum (Figure 4b). The 5 μm elongation applied using the fiber stretcher corresponds to a phase variation in the order of mrad at 10 GHz, which can be easily detected as shown in Figure 4. It is important to note that the 10 Hz stimuli can be clearly distinguished, despite the substantial ambient noise accumulated along the 30 km fiber deployed in a densely populated area of Athens.

### 3.2. Phase Noise Analysis and Comparison to Optical Interferometry

The phase noise analysis, focusing on the properties of the oscillator—whether microwave or optical—is crucial for this specific application. The combination of long distances and the need to detect low-frequency events, such as earthquakes or tsunamis, makes this analysis essential for estimating the performance limits of systems relying on different oscillators. Regarding MFFI, the oscillator is a typical PLL with a phase noise PSD provided by the following expression [18]:

(5) $$S_{PLL,\;dB}\left(f\right)=-128+10\log_{10}\;\left(\frac{10kHz}{f}\right)+20\log_{10}\left(\frac{f_{RF}}{1GHz}\right)$$

The overall system is also affected by the 1/*f* Flicker noise induced by the I/Q mixer at the output, which has been measured and found to be around 2 × 10^−9^/*f* in the low-frequency regime. The noise floor at high frequencies is also determined by the white noise PSD of ASE provided in (4). Hence, the overall phase noise PSD of the MFFI system, ignoring the phase noise of the fiber, which is related to the real signals that the interferometric system seeks to capture, is provided by the following expression:

(6) $$S_{\varphi ,MFFI}(f)=S_{\varphi ,PLL}(f){|1-\exp\left(-i2\pi f{Τ}_{d}\right)|}^{2}+S_{\varphi ,ASE,MFFI}\left(f\right)+2\times 10^{-9}/f$$

In the case of OFI, a high-performance fiber laser is used. The frequency noise PSD of a high-performance laser such as E15, X15 from NKT is documented in [19]; therefore, one can easily calculate and approximate the phase noise PSD of the source. For E15, the frequency noise takes values ranging from 106 Hz^2^/Hz at 10 Hz to 100 Hz^2^/Hz at 20 kHz. The X15 model has better performance with a frequency noise of 300 Hz^2^/Hz at 10 Hz [19]. Using these values, one can provide a rough approximation of the frequency noise PSD, which seems to have a 1/*f*^2^ dependence and a floor of 100 Hz^2^/Hz at high frequencies. The phase noise PSD of the laser is easily calculated using the following expression: $S_{\varphi ,Laser}\left(f\right)=\frac{S_{f,Laser}\left(f\right)}{f^{2}}$. Hence, the final expression of the NKT laser phase noise PSD is $S_{\varphi ,Laser}\left(f\right)=\frac{A}{f^{4}}+\frac{10}{f^{2}}$, where A is estimated to be between 10^4^ and 10^7^, depending on the laser type. Finally, the overall noise of the optical interferometer is provided by the following equation:

(7) $$S_{\varphi ,OFI}(f)=S_{\varphi ,Laser}(f){|1-\exp\left(-i2\pi f{Τ}_{d}\right)|}^{2}+S_{\varphi ,ASE,OFI}\left(f\right)$$

**Figure 5:**
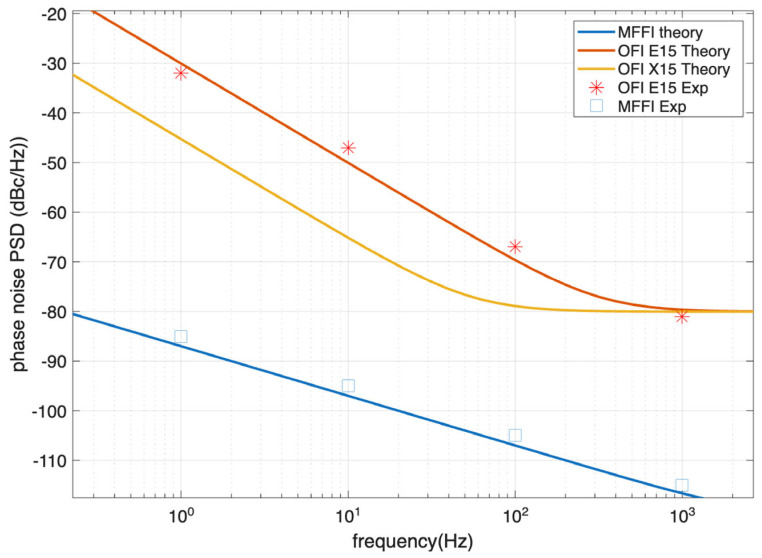
Phase noise PSD of MFFI and OFI based on Equations (4) and (5) and experimental data for an interferometer of 1 km path difference. The experimental data of MFFI are measured by us and the experimental characterization of E15 can be found in [19].

Figure 5 shows how the estimated phase noise from these expressions compares to the measured PSD of MFFI of the new prototype and to the measured phase noise PSD of E15 in interferometric configurations [19]. In this example, the interferometer path difference is 1 km, and the ASE noise is neglected. One can see that there is a strong agreement between theory and experiment. Since X15 provides better noise performance, in the analyses that follows we consider this as the reference source for the OFI systems considered for use in submarine cables.

Then, we investigated the performance of each interrogation technique in a long-haul link at two amplifier spacings, *L_span_* = 50 km and 100 km, respectively. In optical interferometry, the phase that is measured is proportional to the interrogating frequency, as discussed in the beginning of Section 2. Hence, the phase changes measured by OFI are governed by *f_opt_* ~ 190 THz (1550 nm), whilst MFFI has a resolution of *f_RF_* ~ 10 GHz. Hence, the sensitivity advantage of OFI over MFFI is $S_{adv}={\left(\frac{f_{opt}}{f_{RF}}\right)}^{2},\;$which is around 85.5 dB. In both cases, we consider links in the order of *L_total_* = 3000 km length, which determine the *S_ASE_* in (3), (4). For the calculation of the ASE contribution, we consider *n_sp_* = 2, and *P_S_* = −20 dBm. This value is low in order to take into account the effect of the high-loss loopback (−30 dB) in the optical SNR. Here, it must be noted that the sensing signal will be at that low level only for the return path; hence, the ASE noise is overestimated here by a factor of 2. Moreover, the S_ASE_ here is different per span; however, in this analysis, we consider the worst-case scenario of the last span, which is the one that experiences the noise of all amplifiers in the line. The number of amplifiers covering the full link is equal to $N_{amp}=\frac{2L_{total}}{L_{span}}$ as the sensing signal is looped back, as depicted in Figure 1a. The modulation depth for MFFI is considered to be *m* = 0.2. While ASE noise is accumulated in each loop, the effect of the oscillator’s noise must be considered per span as the interferometer measures phase changes over the *i*-th span that can be calculated as ${\Delta \varphi }_{i}=\varphi_{i}-\varphi_{i-1}$, as explained in Section 2.

**Figure 6:**
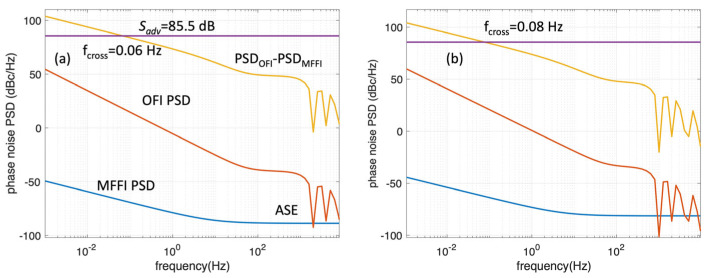
Phase noise PSD of MFFI (blue) and OFI (red) at (**a**) L_span_ = 50 km and (**b**) L_span_ = 100 km. The yellow line corresponds to the difference of OFI PSD minus MFFI PSD. The purple line shows *S_adv_* ~85.5 dB. It becomes clear that OFI has an advantage at frequencies exceeding 0.1 Hz, whilst MFFI can provide better sensitivity at the low-frequency regime. *f_cross_* corresponds to the frequency value for which both systems have similar levels of sensitivity.

As shown in Figure 6, the ASE noise is mostly significant only for MFFI and at frequencies exceeding 100 Hz. At 50 km spacing, the ASE noise induces a constant phase noise PSD of −88 dBc/Hz at MFFI and −108 dBc/Hz for OFI. At 100 km spacing, the noise floor shifts to −81 dBc/Hz and −101 dBc/Hz, respectively. The 20 dB advantage of OFI is related to the scaling factor of *m*^2^/4 that affects MFFI. Despite the low OSNR, this noise term does not affect the low-frequency regime (<1 Hz) that is the main target of geophysical monitoring in the ocean. MFFI has a significantly lower noise PSD than OFI in this regime, which surpass *S_adv_* ~85.5 dB at frequencies below 0.1 Hz, as depicted in both Figure 6a and Figure 6b. The frequency *f_cross_* shows exactly the point where both systems have equivalent sensitivity as, at this point, the *S_adv_* = 85.5 dB advantage of OFI in sensitivity is completely compensated by the 85.5 dB advantage of MFFI in phase noise PSD. The theoretical model estimates *f_cross_* ~0.06 Hz for 50 km and *f_cross_* ~0.08 Hz for 100 km.

## 4. Discussion

Regarding the experimental results, the observed signal—due to the low optical power of the photodetector—exhibited a maximum swing of 100 mVpp. With the addition of amplifiers in both the optical and electrical domains, the interrogator is ex-pected to utilize the full dynamic range of the ADC (3 Vpp) and detect sub-milliradian phase variations. These estimates can be readily obtained by evaluating the RMS phase noise over the bandwidth of interest. Based on Figure 6b, for instance, phase noise PSD is approximately −50 dBc/Hz at 0.01 Hz and approximately −70 dBc/Hz at 1 Hz for the MFFI system. By integrating the phase noise in this window, one can easily find that the variance of phase noise is lower than 10^−6^ rad^2^; hence, the RMS phase noise will be less than 10^−3^ rad (mrad). Adequate amplification ensures that the signal will not be affected by photodetection and quantization noise. This helps preserve a low noise figure across all spans, despite the 30 dB loss introduced by the high-loss loopback. The minimum detectable strain can be found if RMS phase is converted to RMS displacement using the following expression: $\Delta L_{RMS}=\frac{c}{\omega_{RF}n_{g}}\Delta \varphi_{RMS}$. The displacement that can be measured is below 3 μm for an interferometer of 200 km, assuming $\Delta \varphi_{RMS}<mrad$. For this displacement at 200 km, the minimum detectable strain is below 1.5 × 10^−11^ (sub nanostrain).

The low-frequency noise shown in Figure 4 (below 10 Hz) arises from environmental perturbations affecting the fibers, such as temperature fluctuations and acoustic noise pickup. As previously noted, the installed fiber is significantly affected by anthropogenic noise, as it is located in a densely populated area of Athens. Therefore, this contribution does not originate from the source itself, but rather reflects fiber-induced noise detected by the interrogator.

Based on the theoretical analysis, amplified spontaneous emission (ASE) noise is not expected to significantly affect the system sensitivity at the low frequencies relevant for geophysical monitoring. To enhance sensitivity, it is important to ensure a sufficiently strong signal prior to photodetection; therefore, a pre-amplifier will be required in a real-world field deployment. Depending on the stability of the laser source used in transoceanic experiments, it is important to note that *f_cross_* will vary. Here, by comparing a typical PLL with X15 laser from NKT, this value is estimated to between 0.01 and 0.1 Hz. Regardless of the exact crossover point, both systems appear to become broadly equivalent in this regime. This suggests that MFFI is a strong candidate for oceanic geophysical monitoring (e.g., ocean swells, tides, and large-scale geophysical signals) as well as temperature measurements. In contrast, at frequencies above 10 Hz, where anthropogenic noise (e.g., vessels and machinery) and marine mammal activity (such as whales and dolphins) are present, OFI enhances sensitivity by at least 20 dB. MFFI relies on simple optoelectronic devices and data acquisition takes place at very low sampling rates (1 Msps) which vastly reduces the cost and consumption of the device and paves the way for the real-time processing of data for early warning purposes.

In order to avoid any crosstalk between different loops in such a long-haul transmission scenario, a gating mechanism with a very high on–off switching (>50 dB) must be chosen. Acousto-optic modulators are a good candidate, as reported in Section 2. Another choice could be the use of a semiconductor optical amplifier which is known to be a very high-fidelity switch and can also act as an amplifier-booster of the signal. It should be noted that the gating mechanism cannot prevent noise from entering through in-line amplifiers; however, strong gating effectively eliminates coherent crosstalk, which has a more detrimental impact on signal fidelity. Issues related to power fading caused by chromatic dispersion can be addressed using diverse techniques including carrier-suppressed dual-sideband modulation [20], single sideband modulation [21] or an interrogation algorithm where multiple RF frequencies are sent in the link in parallel. Such a scheme could also increase the interrogated bandwidth which is now limited by *T_rep_*. For instance, for *L* = 3000 km considered in this study, *T_rep_* = 30 ms, which corresponds to *f_s_* < 33 Hz or an observation bandwidth of 17 Hz, which is marginally adequate for complete geophysical monitoring. One technique to further increase the observation bandwidth is to transmit multiple pulses in separate time slots using distinct RF frequencies, which also enhances frequency diversity to help mitigate fading. Similar techniques are adopted for DAS systems as well [22]. The ratio between *T_rep_* and *T_w_* determines the SNR of the signal as the average power is proportional to *T_w_/T_rep_*. Hence, as the fiber length increases, the SNR decreases due to the combined effects of greater accumulated noise and lower average optical power resulting from a reduced duty cycle. This limitation can be mitigated through the use of the multi-frequency transmission technique described above. Hence, a two-frequency MFFI system that transmits pulses in different time slots can simultaneously improve the sampling rate, as well as the system’s tolerance to fading and noise.

The impact of the MFFI wavelength on WDM traffic in joint communication and sensing deployments is expected to be minimal. Unlike DAS, which relies on scattering, the transmission-based nature of MFFI reduces the need for high launch power; conse-quently, the average power of MFFI can be lower than that of individual WDM channels. Therefore, its presence is not expected to significantly disturb co-propagating WDM communication channels. Nonlinear transmission effects, such as cross-phase modulation (XPM), may still represent a potential source of crosstalk, particularly in terms of how the MFFI signal is influenced by a large number of WDM channels. However, if the ap-plication targets bandwidths below a few hertz, as in geophysical monitoring, XPM is unlikely to pose a critical issue. At such low bandwidths (<10 Hz), the relative intensity noise of co-propagating channels is expected to exceed 60–70 dB, considering that the OSNR for multi-GHz telecom channels carrying high-order modulation formats must typically be above 15–20 dB to achieve acceptable bit-error-rate performance. The stochastic part of the nonlinear phase shift induced by each WDM channel to the MFFI sensing wavelength is proportional to 2*ΔPγL_eff_*, where *ΔP* corresponds to power variations per channel (10^−5^ of average power at Hz level bandwidths in a pessimistic consideration), *γ* is the nonlinear parameter of the fiber (1.3 W^−1^km^−1^ for single-mode fibers) and *L_eff_* is the effective length of the fiber taking into account losses which (20 km for single-mode fiber) [23]. For *N* = 40 WDM channels and a link consisting of *N_amp_* = 30 amplifiers (100 km spacing, *L* = 3000 km), the overall phase variation due to XPM is, at maximum, 2*NN_amp_ΔPγL_eff_*. A simple calculation indicates that XPM-induced noise is approximately 0.5 mrad, which is close to the sensitivity of our system. Furthermore, because MFFI employs direct detection, XPM effects are significantly reduced prior to the downconversion stage where phase noise is measured. Subsequent studies should clearly focus on how such an interrogation technique could affect telecom traffic and vice versa in dense WDM scenarios.

## 5. Conclusions

We presented a variant of MFFI operating at 10 GHz that enables coarse localization at span resolution by leveraging periodic loopbacks commonly deployed in transoceanic cables for amplifier monitoring. Clear identification of Hz-level events per loop has been experimentally demonstrated. We also provided a theoretical analysis showing that, at low frequencies (<0.1 Hz), MFFI is nearly equivalent to OFI in terms of sensitivity, when accounting for the noise characteristics of state-of-the-art optical sources. Given the sim-plicity and satisfactory accuracy of the MFFI technique, the proposed scheme could serve as an alternative approach for interrogating transoceanic cables, potentially enabling early-warning systems for natural hazards in the deep ocean.

## Data Availability

The original contributions presented in this study are included in the article. Further inquiries can be directed to the corresponding author.
